# Supplementary material for: Patterns of Intron Gain and Loss in Fungi
Source: PLoS Biol. 2004 Nov 30;2(12):e422. doi: 10.1371/journal.pbio.0020422 (PMC532390; doi:10.1371/journal.pbio.0020422)
Supplement: Table S1 — Also available at http://genes.mit.edu/NielsenEtAl/. (4.3 MB ZIP). [file pbio.0020422.st001.zip › NielsenEtAl/html/864.html]

AN7030.1.NCU06593.1.MG01702.1.FG05261.1


```
 CLUSTAL W (1.82) Multiple Sequence Alignments - Introns Inserted


Sequence 1: MG01702.1	621 aa
Sequence 2: FG05261.1	590 aa
Sequence 3: NCU06593.1	576 aa
Sequence 4: AN7030.1	616 aa
Alignment Length: 664 aa
Number Identitical Residues: 268 aa
Alignment Score (without introns) 12814


MG01702.1 	----MK0ALRRSIKG-DKDKQPQ------------ISIAPKSAVAIVPPKK0VIRALYDY
NCU06593.1	MSRGFK~ALRRSIKGGEKDSKPH------------ITIQPKSAVAIVPPKK0VIKALYDY
FG05261.1 	----MK0ALRRSIKG-DKDKGP---------------VTPKSAVAIVPPKK0VIRALYDY
AN7030.1  	----MK0ALRRSLKG-EKDSKPHHHHQHHHQQHHHLSITPKSAVAILPPKK0VIKALYDY
          	    :* *****:** :**. *    .   ..    :: *******:**** **:*****

MG01702.1 	EAQ--STQELSFSRGDFFHVIGRENDTDWYEACNPALPDARGLVPVAFFQALGRTERDSA
NCU06593.1	EAQ--NAQELSFSRGDFFHVIGRENDPEWYEACNPALPDARGLVPVAYFQALGRTERDSG
FG05261.1 	EAR--SSQELSFSRGDFFHVIGRENDDDWYEACNPALPDARGLVPVTFFQALGRTERDSA
AN7030.1  	QPEPGNTQELAFSKGDFFHVISREDDSDWYEACNPLIPTARGLVPVTFFEVIGKTQRDSS
          	:.....:***:**:*******.**:* :******* :* *******::*:.:*:*:***.

MG01702.1 	QSDTSVTRTSAPVPSKNPDHDSGYAETSAPVPS--TPGFS----------NRDSKS-GKS
NCU06593.1	QSQTGSLG------LKTVDSDSGYGEGSAASPP--GANAA----------NRMSKTPGKN
FG05261.1 	QSDGGQP----PSPPKNPDHDSGYSESPAMHPA--PAVMSPTTNSAPQGHQRNSKSVGKT
AN7030.1  	GSIDLGK--------KKEPHDSGFADRATSSGSEFGSGSSKSYQSHPAFPRTSMMGKAGG
          	 *             *.   ***:.: .:   ..  .  : : .: .   .      .  

MG01702.1 	GAMVYGIVMYDFQAERADELQAQAGEAIIVIAQSNPEWFVAKPIGRLGGPGLIPVSFIEI
NCU06593.1	GAMVYGVVLYDFQAERADELEAKQGEAIIVIAQSNPEWFVAKPIGRLGGPGLIPVSFVEI
FG05261.1 	GAMVYGIVMYDFAAERADELEAKAGEAIIVIAQSNPEWFVAKPIGRLGGPGLIPVSFVEI
AN7030.1  	GAMVYGVVQYDFQAERPDELDAKAGEAIIVIAQSNPEWFVAKPIGRLGGPGLIPVSFIEL
          	******:* *** ***.***:*: *********************************:*:

MG01702.1 	RDMASNTPVPDPEGAVRRAGIPRVEEWKKMAAEYKNSSITLGKFEVGGAPQQ-QAIEQGM
NCU06593.1	RDMASNTPVPNPHDAIRKAGIPKVEEWKKMAAEYKNSSITLGKFDVGGGGGGQPPLDQAM
FG05261.1 	RDMASDKAIQNPGDAIKRAGVPKVEEWKKMAAEYKNSSITLGKFEGGGPGQPGQGIEQGM
AN7030.1  	RDMQSGQAVADPLEAVRRAGVPKVEEWKKMTAEYKNSSISLGKIETTG---SMQSVTSGM
          	*** *. .: :*  *:::**:*:*******:********:***::  *       : ..*

MG01702.1 	DRMSLQPTDSRSSQGQLNGKL0QQQQQPQPQPQQQQQQQQPQ~QP~GYNSQRNTQQQ~AM
NCU06593.1	NRLTLQG--------------~--------------------~-S~ARLSQQTTHTQ0QQ
FG05261.1 	ERMSIQ---------------~QQQHSRQPS-------QNGT0QS~GYASQPRTSQQ~PQ
AN7030.1  	ERMSVRQS-------------~-ADHVSHMS-------QNGH~AN0AYHNRNASKAS~IA
          	:*:::: :                .     .       ..      .  .:  :  .   

MG01702.1 	QPQNAYPTPKPSTQLYAPVQASIPRYCFAEEKYWFVIEAILEDGRKYELSRYYEDFYDFQ
NCU06593.1	IQAQGQSYSRPTSTLYAPIAARVPRYCFAEDKYWFVIEAELEDGRCFELSRYYEDFYDFQ
FG05261.1 	PHQS--QVQKPAAPLHAPLEARIPRYCFAEDKYWFVIEAVLEDGRQWELSRYYEDFYDFQ
AN7030.1  	PSTHQQPPPHSQQPLVAPIAASIPRYCFDNDKYWYIIEVKMEDGRCWELSRYYHDFYDFQ
          	         :.   * **: * :***** ::***::**. :**** :******.******

MG01702.1 	IALLTEFPAEAGNTGTQKRTLPYMPGPVNYVTDAITEGRLHNLDAYVKNLLAQPTYISRC
NCU06593.1	IALLTEFPLEAGTTGGKQRTLPYMPGPVNYVTDAITEGRRHNLDSYVKSLLEQPPHISRC
FG05261.1 	IALLTEFPAEAGNTGTQKRTLPYMPGPVNYVTDAITEGRLHNLDAYVKNMLNQPPYISRC
AN7030.1  	IALLTQFEEEAGNRG-KPRTLPFMPGPVAHVTDAISDGRRHNLDEYIKKLLAMPPHITRC
          	*****:*  ***. * : ****:***** :*****::** **** *:*.:*  *.:*:**

MG01702.1 	NLVKQFFAPREGDYEMDANGEEDEYRMSGGSQQSSPDSPNGGISQQSSRNNLSSGGYNNN
NCU06593.1	NLVKQFFSPREGDYEIDPSVAREEYRFSGGSGPSS-DSPADGLSLQSTAGNRYSAGLG--
FG05261.1 	NLVRQFFAPREGDYEIDPNDNENEYRLSQGSQLSSMESPAGG-----SQNNLNGSGYG--
AN7030.1  	ALVRQLFAPRPGDFEIDPSAFGEDARFSGGSQHSSGHELSPSASRQSSQQQNGPSERSSH
          	 **:*:*:** **:*:*..   :: *:* **  ** ..   . : .::  :   .  .. 

MG01702.1 	NLAPSQQRGLSAQPPMMRQASS---LSQP----SQASLSPGIPQAG---AFMKVKLSYNG
NCU06593.1	--VPQRQ----LSIGNGQQIPG---PGQA----SQTSLSPAIMQGG---SQMKVKLHFNG
FG05261.1 	-LAPS-------HPDMQTQASS---LSQP----SQASLGGGMQAPSQPPSAMKIKMYFNG
AN7030.1  	SHSHSHS-----HSHQRSQTSSSIPNGKPPMVRQQSSLTQVSTSSG---GALKVKVNFQD
          	.   . .           * ..: . .:..   .*:**       .   . :*:*: ::.

MG01702.1 	DLIAIRVPSDIQFRELFDKITERLRIQPGEEVQLSYKDDITNGKLALMNDQDLIYAMQRN
NCU06593.1	DIIAIRVPTDIPFQALHDKILDRLKIPQGEDVQLSYKDDASGEKPPLMNDEDLDIALQRN
FG05261.1 	DLIAIRVPTDISFQALYDKICDRLKIPANEEIQLFYKDEPTGDKPSMISDNDLDFALQRN
AN7030.1  	DLIAIRVPSDINVQQLKEKLMDRLKIN--DEIVVQYKDEASGAYVDLISDSDLDTAIQRN
          	*:******:** .: * :*: :**:*   ::: : ***: :.    ::.*.**  *:***

MG01702.1 	EKLLFYVDHI
NCU06593.1	EKLLVYVE--
FG05261.1 	EKLLLYVEAV
AN7030.1  	SKLTLYVGLA
          	.** .**
```
